# The genome sequence of the garden grass-veneer, *Chrysoteuchia culmella* (Linnaeus, 1758)

**DOI:** 10.12688/wellcomeopenres.18107.1

**Published:** 2022-10-05

**Authors:** Douglas Boyes, University of Oxford and Wytham Woods Genome Acquisition Lab, Darwin Tree of Life Barcoding collective, Wellcome Sanger Institute Tree of Life programme, Wellcome Sanger Institute Scientific Operations: DNA Pipelines collective, Tree of Life Core Informatics collective, Louis Parkerson, Darwin Tree of Life Consortium

**Affiliations:** 1 UK Centre for Ecology and Hydrology, Wallingford, Oxfordshire, UK; 2 Independent researcher, Norwich, Norfolk, UK

**Keywords:** Chrysoteuchia culmella, garden grass-veneer, genome sequence, chromosomal, Crambidae

## Abstract

We present a genome assembly from an individual male *Chrysoteuchia culmella* (the garden grass-veneer; Arthropoda; Insecta; Lepidoptera; Crambidae). The genome sequence is 645 megabases in span. The majority of the assembly (99.81%) is scaffolded into 31 chromosomal pseudomolecules with the Z sex chromosome assembled. The complete mitochondrial genome was also assembled and is 15.4 kilobases in length. Gene annotation of this assembly on Ensembl has identified 21,251 protein coding genes.

## Species taxonomy

Eukaryota; Metazoa; Ecdysozoa; Arthropoda; Hexapoda; Insecta; Pterygota; Neoptera; Endopterygota; Lepidoptera; Glossata; Ditrysia; Pyraloidea; Crambidae; Crambinae; *Chrysoteuchia*; *Chrysoteuchia culmella* (Linnaeus, 1758) (NCBI:txid1594250).

## Background

The garden grass-veneer, *Chrysoteuchia culmella* (Linnaeus, 1758), is a micro moth of the Crambinae subfamily. It is common in grassland, rough meadows and gardens throughout much of Europe, including the British Isles ( “Chrysoteuchia Culmella (Linnaeus, 1758)” 2010). It is recognised by its angled subterminal line, golden metallic cilia and size, with a wingspan of 20–24mm ( “Chrysoteuchia Culmella (Garden Grass-Veneer)” 2012). The eggs are laid on various grasses and, after hatching, the larvae feed from September to April on the stem bases of grasses. After pupating in May from a cocoon near the ground, the species is on the wing from mid-May to mid-September (and occasionally until late October). During this time, it can be readily disturbed from grasses during the day and attracted to light during the night ( Langmaid *et al.*, 2018). *C. culmella* larvae are frequent hosts of the endoparasitic larvae of *Eriothrix rufomaculata*, a parasitoid species of fly ( Paston & Rotheray, 2009). We present a complete genome assembly for *C. culmella* as part of the Darwin Tree of Life project, Wellcome Sanger Institute, aiming to sequence the genomes of 70,000 species of eukaryotic organisms in Britain and Ireland.

## Genome sequence report

**Figure 1.**
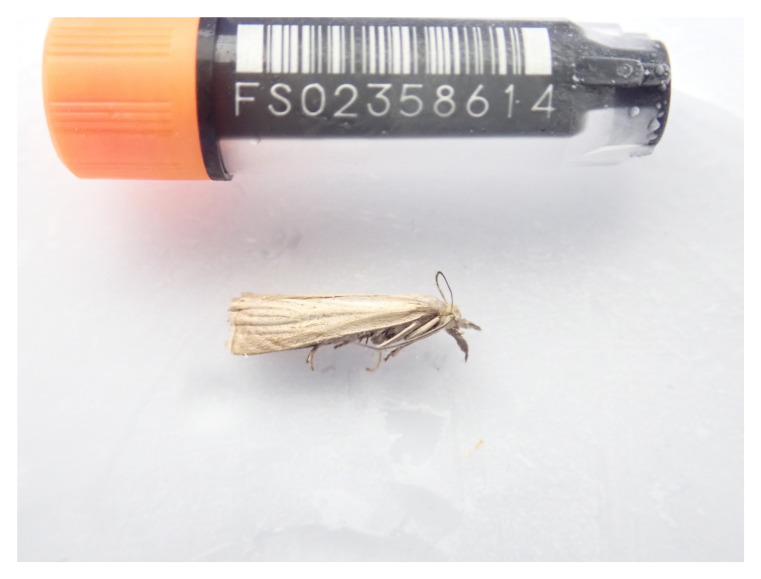
Image of the *Chrysoteuchia culmella* specimen taken prior to preservation and processing.

The genome was sequenced from a single male *C. culmella* (ilChrCulm1) collected from Wytham Woods, Berkshire, UK ( Figure 1). A total of 43-fold coverage in Pacific Biosciences single-molecule HiFi long reads and 56-fold coverage in 10X Genomics read clouds were generated. Primary assembly contigs were scaffolded with chromosome conformation Hi-C data. Manual assembly curation corrected 18 missing/misjoins and removed 2 haplotypic duplications, reducing the assembly size by 0.64% and the scaffold number by 17.39%, and increasing the scaffold N50 by 4.73%.

**Figure 2.**
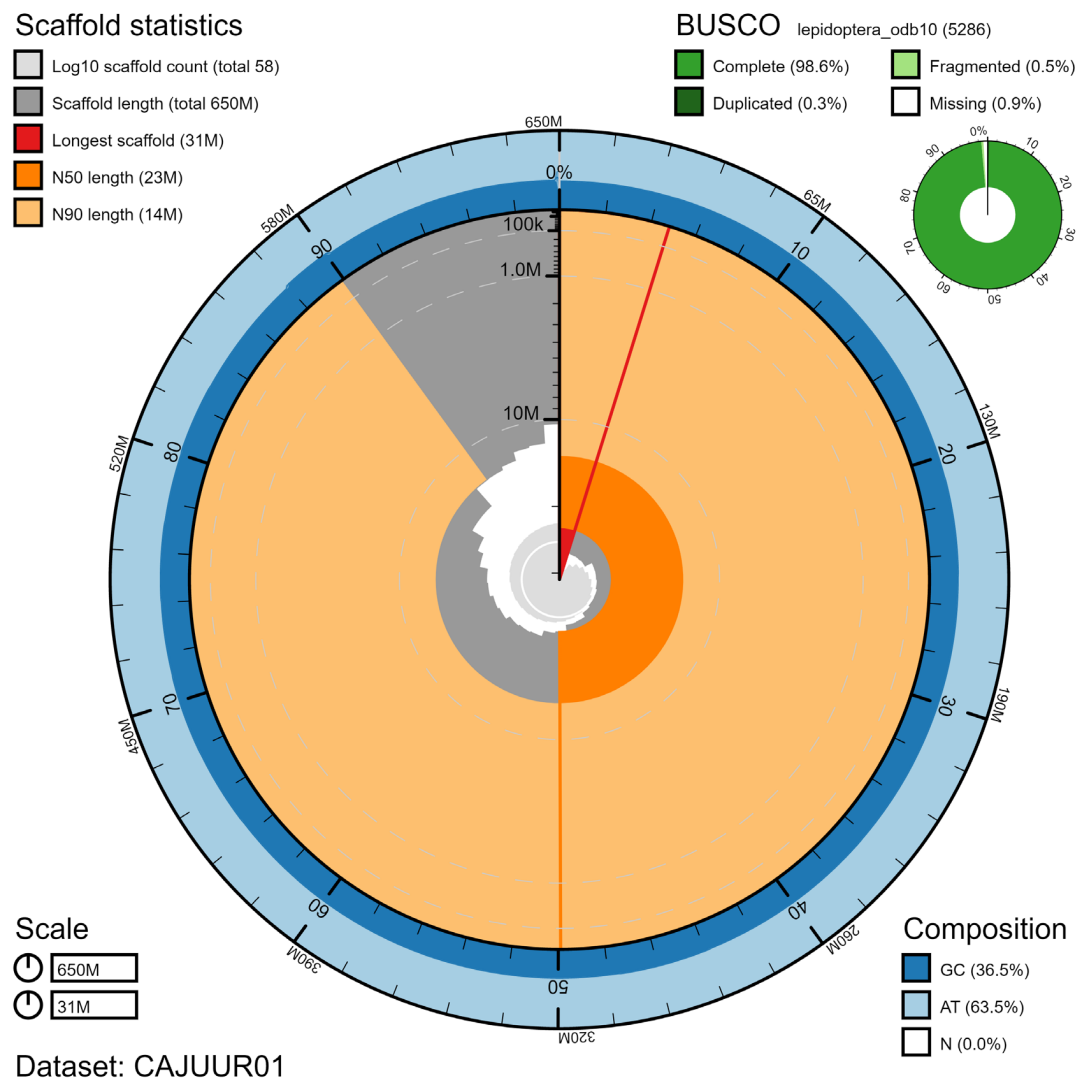
Genome assembly of *Chrysoteuchia culmella*, ilChrCulm1.1: metrics. The BlobToolKit Snailplot shows N50 metrics and BUSCO gene completeness. The main plot is divided into 1,000 size-ordered bins around the circumference with each bin representing 0.1% of the 645,226,651 bp assembly. The distribution of chromosome lengths is shown in dark grey with the plot radius scaled to the longest chromosome present in the assembly (31,066,094 bp, shown in red). Orange and pale-orange arcs show the N50 and N90 chromosome lengths (22,805,156 and 13,759,558 bp), respectively. The pale grey spiral shows the cumulative chromosome count on a log scale with white scale lines showing successive orders of magnitude. The blue and pale-blue area around the outside of the plot shows the distribution of GC, AT and N percentages in the same bins as the inner plot. A summary of complete, fragmented, duplicated and missing BUSCO genes in the lepidoptera_odb10 set is shown in the top right. An interactive version of this figure is available at https://blobtoolkit.genomehubs.org/view/ilChrCulm1.1/dataset/CAJUUR01/snail.

The final assembly has a total length of 645 Mb in 57 sequence scaffolds with a scaffold N50 of 22.8 Mb ( Table 1). The majority, 99.81%, of the assembly sequence was assigned to 31 chromosomal-level scaffolds, representing 30 autosomes (numbered by sequence length) and the Z sex chromosome ( Figure 2– Figure 5; Table 2).

**Table 1.** Genome data for *Chrysoteuchia culmella*, ilChrCulm1.1.

*Project accession data*	
Assembly identifier	ilChrCulm1.1
Species	*Chrysoteuchia culmella*
Specimen	ilChrCulm1 (genome assembly); ilChrCulm2 (Hi-C, RNA-Seq)
NCBI taxonomy ID	1594250
BioProject	PRJEB45126
BioSample ID	SAMEA7701502
Isolate information	Male, whole organism (ilChrCulm1); Whole organism tissue, unknown sex (ilChrCulm2)
*Raw data accessions*	
PacificBiosciences SEQUEL II	ERR6406210
10X Genomics Illumina	ERR6054801-ERR6054804
Hi-C Illumina	ERR6054805
PolyA RNA-Seq Illumina	ERR9434977
*Genome assembly*	
Assembly accession	GCA_910589605.1
*Accession of alternate haplotype*	GCA_910589405.1
Span (Mb)	645
Number of contigs	89
Contig N50 length (Mb)	16.4
Number of scaffolds	57
Scaffold N50 length (Mb)	22.8
Longest scaffold (Mb)	26.47
BUSCO * genome score	C:98.6%[S:98.3%,D:0.3%], F:0.5%,M:0.9%,n:5,286

*BUSCO scores based on the lepidoptera_odb10 BUSCO set using v5.3.2. C= complete [S= single copy, D=duplicated], F=fragmented, M=missing, n=number of orthologues in comparison. A full set of BUSCO scores is available at https://blobtoolkit.genomehubs.org/view/ilChrCulm1.1/dataset/CAJUUR01.1/busco.

**Figure 3.**
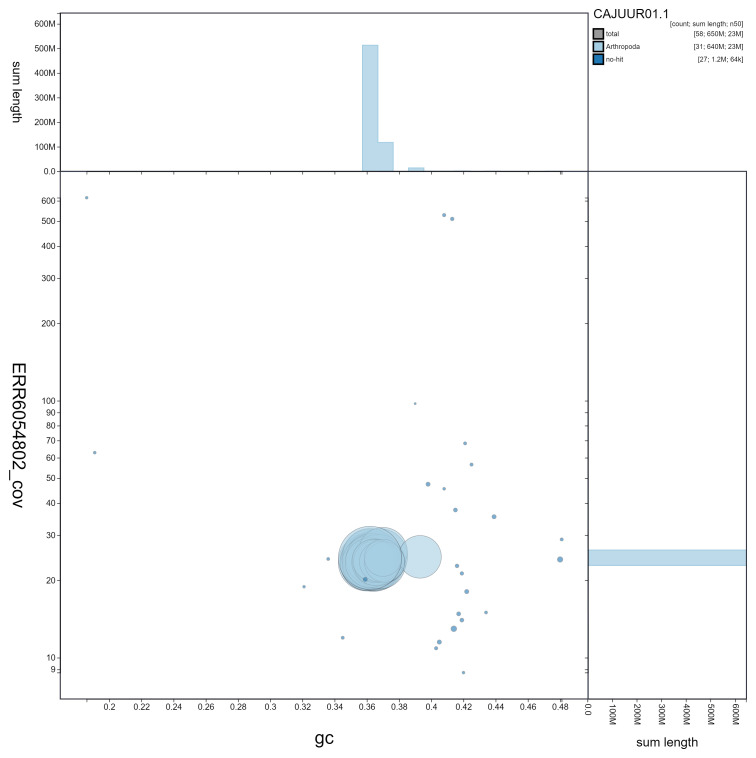
Genome assembly of *Chrysoteuchia culmella*, ilChrCulm1.1: GC coverage. BlobToolKit GC-coverage plot. Scaffolds are coloured by phylum. Circles are sized in proportion to scaffold length. Histograms show the distribution of scaffold length sum along each axis. An interactive version of this figure is available at https://blobtoolkit.genomehubs.org/view/ilChrCulm1.1/dataset/CAJUUR01.1/blob.

**Figure 4.**
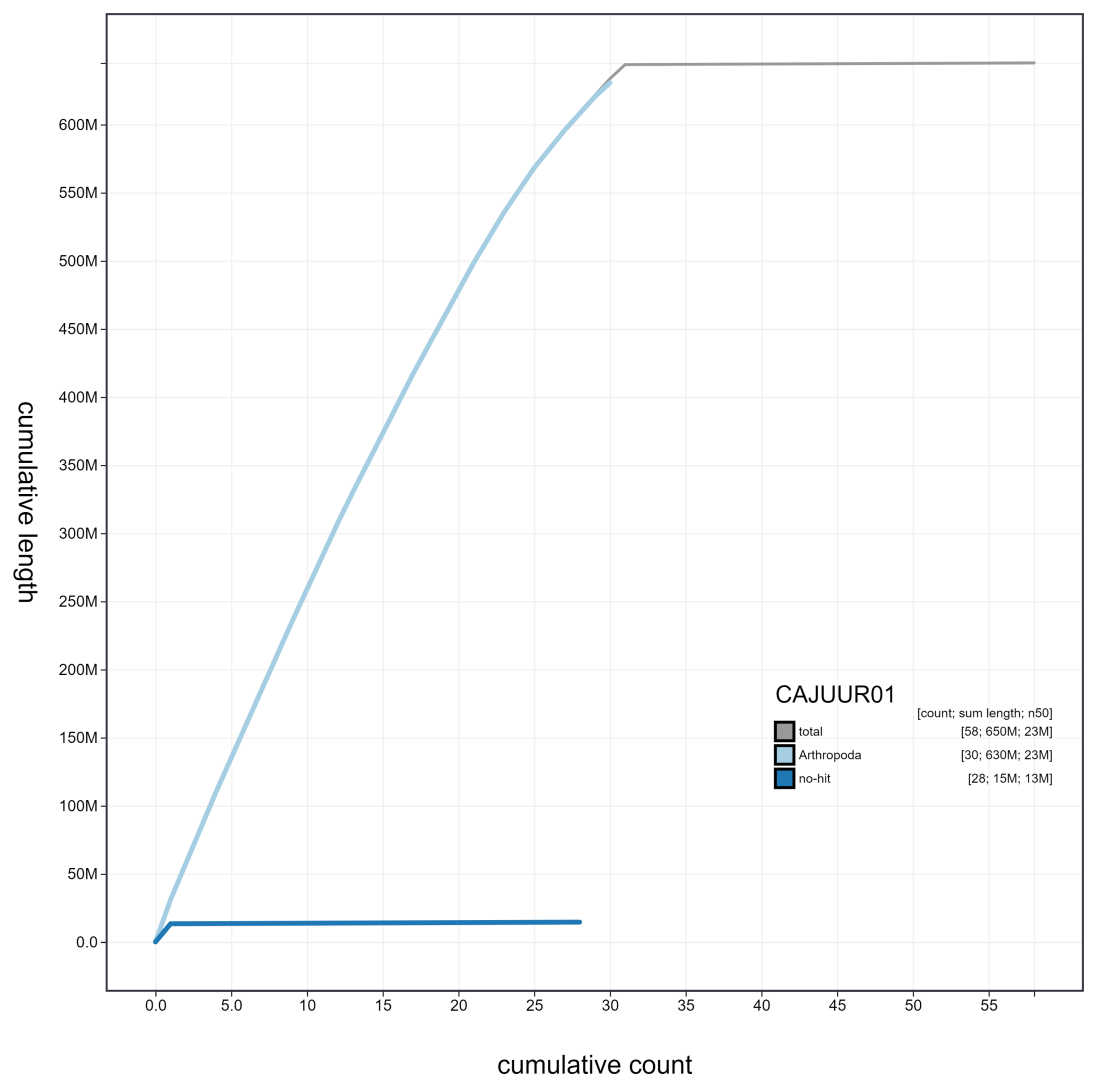
Genome assembly of *Chrysoteuchia culmella*, ilChrCulm1.1: cumulative sequence. BlobToolKit cumulative sequence plot. The grey line shows cumulative length for all scaffolds. Coloured lines show cumulative lengths of scaffolds assigned to each phylum using the buscogenes taxrule. An interactive version of this figure is available at https://blobtoolkit.genomehubs.org/view/ilChrCulm1.1/dataset/CAJUUR01.1/cumulative.

**Figure 5.**
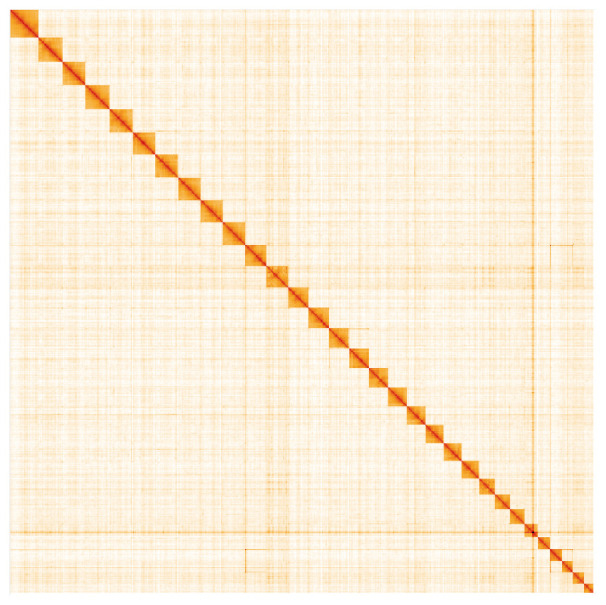
Genome assembly of *Chrysoteuchia culmella*, ilChrCulm1.1: Hi-C contact map. Hi-C contact map of the ilChrCulm1.1 assembly, visualised in HiGlass. Chromosomes are arranged in size order from left to right and top to bottom. The interactive Hi-C map can be viewed at https://genome-note-higlass.tol.sanger.ac.uk/l/?d=PizMaC2sTVqZKsjMxz2y9A.

**Table 2.** Chromosomal pseudomolecules in the genome assembly of *Chrysoteuchia culmella*, ilChrCulm1.1.

INSDC accession	Chromosome	Size (Mb)	GC%
OU342642.1	1	26.47	36.5
OU342643.1	2	26.39	36.4
OU342644.1	3	26.29	36.2
OU342645.1	4	25.12	36
OU342646.1	5	25.07	36.4
OU342647.1	6	24.95	36.1
OU342648.1	7	24.75	36.1
OU342649.1	8	24.7	36.2
OU342650.1	9	24.46	36.4
OU342651.1	10	23.8	36.3
OU342652.1	11	23.69	36.8
OU342653.1	12	22.81	36.2
OU342654.1	13	22.6	36.2
OU342655.1	14	21.77	36.4
OU342656.1	15	21.63	36.3
OU342657.1	16	21.42	36.1
OU342658.1	17	20.79	36.5
OU342659.1	18	20.54	36.8
OU342660.1	19	20.08	36.3
OU342661.1	20	19.95	36.7
OU342662.1	21	18.88	36.2
OU342663.1	22	18.12	37
OU342664.1	23	16.43	36.5
OU342665.1	24	16.42	36.6
OU342666.1	25	13.76	39.3
OU342667.1	26	13.71	36.5
OU342668.1	27	13.34	36.4
OU342669.1	28	12.4	36.8
OU342670.1	29	12.24	37.1
OU342671.1	30	10.36	37
OU342641.1	Z	31.07	36.2
OU342672.1	MT	0.02	18.8
-	Unplaced	1.2	41.4

The assembly has a BUSCO v5.13.2 ( Manni *et al.*, 2021) completeness of 98.6% (single 98.3%, duplicated 0.3%) using the lepidoptera_odb10 reference set (n=5,286). While not fully phased, the assembly deposited is of one haplotype. Contigs corresponding to the second haplotype have also been deposited.

## Genome annotation report

The ilChrCulm1.1 genome has been annotated using the Ensembl rapid annotation pipeline ( Table 1; https://rapid.ensembl.org/Chrysoteuchia_culmella_GCA_910589605.1/). The resulting annotation includes 21,475 transcribed mRNAs from 21,251 protein-coding genes.

## Methods

### Sample acquisition and nucleic acid extraction

Two *C. culmella* specimens (ilChrCulm1, genome assembly; ilChrCulm2, Hi-C and RNA-Seq) were collected using a light trap from Wytham Woods, Berkshire, UK (latitude 51.772, longitude -1.338) by Douglas Boyes (University of Oxford). The specimens were identified by Douglas Boyes snap-frozen on dry ice.

DNA was extracted at the Tree of Life laboratory, Wellcome Sanger Institute. The ilChrCulm1 sample was weighed and dissected on dry ice. Whole organism tissue was disrupted using a Nippi Powermasher fitted with a BioMasher pestle. Fragment size analysis of 0.01–0.5 ng of DNA was then performed using an Agilent FemtoPulse. High molecular weight (HMW) DNA was extracted using the Qiagen MagAttract HMW DNA extraction kit. Low molecular weight DNA was removed from a 200-ng aliquot of extracted DNA using 0.8X AMpure XP purification kit prior to 10X Chromium sequencing; a minimum of 50 ng DNA was submitted for 10X sequencing. HMW DNA was sheared into an average fragment size between 12–20 kb in a Megaruptor 3 system with speed setting 30. Sheared DNA was purified by solid-phase reversible immobilisation using AMPure PB beads with a 1.8X ratio of beads to sample to remove the shorter fragments and concentrate the DNA sample. The concentration of the sheared and purified DNA was assessed using a Nanodrop spectrophotometer and Qubit Fluorometer and Qubit dsDNA High Sensitivity Assay kit. Fragment size distribution was evaluated by running the sample on the FemtoPulse system.

RNA was extracted from whole organism tissue of ilChrCulm2 in the Tree of Life Laboratory at the WSI using TRIzol, according to the manufacturer’s instructions. RNA was then eluted in 50 μl RNAse-free water and its concentration RNA assessed using a Nanodrop spectrophotometer and Qubit Fluorometer using the Qubit RNA Broad-Range (BR) Assay kit. Analysis of the integrity of the RNA was done using Agilent RNA 6000 Pico Kit and Eukaryotic Total RNA assay.

### Sequencing

Pacific Biosciences HiFi circular consensus and 10X Genomics Chromium read cloud sequencing libraries were constructed according to the manufacturers’ instructions. Sequencing was performed by the Scientific Operations core at the Wellcome Sanger Institute on Pacific Biosciences SEQUEL II (HiFi), Illumina NovaSeq 6000 (10X) and Illumina HiSeq 4000 (RNA-Seq) instruments. Hi-C data were generated in the Tree of Life laboratory from remaining tissue of ilChrCulm2 using the Arima v2 kit and sequenced on a NovaSeq 6000 instrument.

### Genome assembly

Assembly was carried out with Hifiasm ( Cheng *et al.*, 2021); haplotypic duplication was identified and removed with purge_dups ( Guan *et al.*, 2020). One round of polishing was performed by aligning 10X Genomics read data to the assembly with longranger align, calling variants with freebayes ( Garrison & Marth, 2012). The assembly was then scaffolded with Hi-C data ( Rao *et al.*, 2014) using SALSA2 ( Ghurye *et al.*, 2019). The assembly was checked for contamination and corrected using the gEVAL system ( Chow *et al.*, 2016) as described previously ( Howe *et al.*, 2021). Manual curation ( Howe *et al.*, 2021) was performed using gEVAL, HiGlass ( Kerpedjiev *et al.*, 2018) and Pretext. The mitochondrial genome was assembled using MitoHiFi ( Uliano-Silva *et al.*, 2021), which performs annotation using MitoFinder ( Allio *et al.*, 2020). The genome was analysed and BUSCO scores generated within the BlobToolKit environment ( Challis *et al.*, 2020). Table 3 contains a list of all software tool versions used, where appropriate.

**Table 3.** Software tools used.

Software tool	Version	Source
Hifiasm	0.14	Cheng *et al.*, 2021
purge_dups	1.2.3	Guan *et al.*, 2020
SALSA2	2.2	Ghurye *et al.*, 2019
longranger align	2.2.2	https://support.10xgenomics.com/ genome-exome/software/pipelines/ latest/advanced/other-pipelines
freebayes	1.3.1-17- gaa2ace8	Garrison & Marth, 2012
MitoHiFi	2.0	Uliano-Silva *et al.*, 2021
HiGlass	1.11.6	Kerpedjiev *et al.*, 2018
PretextView	0.2.x	https://github.com/wtsi-hpag/ PretextView
BlobToolKit	3.2.6	Challis *et al.*, 2020

### Genome annotation

The Ensembl gene annotation system ( Aken *et al.*, 2016) was used to generate annotation for the *Chrysoteuchia culmella* assembly (GCA_910589605.1). Annotation was created primarily through alignment of transcriptomic data to the genome, with gap filling via protein-to-genome alignments of a select set of proteins from UniProt ( UniProt Consortium, 2019).

### Ethics/compliance issues

The materials that have contributed to this genome note have been supplied by a Darwin Tree of Life Partner. The submission of materials by a Darwin Tree of Life Partner is subject to the Darwin Tree of Life Project Sampling Code of Practice. By agreeing with and signing up to the Sampling Code of Practice, the Darwin Tree of Life Partner agrees they will meet the legal and ethical requirements and standards set out within this document in respect of all samples acquired for, and supplied to, the Darwin Tree of Life Project. Each transfer of samples is further undertaken according to a Research Collaboration Agreement or Material Transfer Agreement entered into by the Darwin Tree of Life Partner, Genome Research Limited (operating as the Wellcome Sanger Institute), and in some circumstances other Darwin Tree of Life collaborators.

## Data Availability

European Nucleotide Archive: Chrysoteuchia culmella (garden grass-veneer). Accession number PRJEB45126; https://identifiers.org/ena.embl/PRJEB45126. The genome sequence is released openly for reuse. The *C. culmella* genome sequencing initiative is part of the Darwin Tree of Life (DToL) project. All raw sequence data and the assembly have been deposited in INSDC databases. Raw data and assembly accession identifiers are reported in Table 1. Members of the University of Oxford and Wytham Woods Genome Acquisition Lab are listed here: https://doi.org/10.5281/zenodo.6418202. Members of the Darwin Tree of Life Barcoding collective are listed here: https://doi.org/10.5281/zenodo.6418156. Members of the Wellcome Sanger Institute Tree of Life programme are listed here: https://doi.org/10.5281/zenodo.6866293. Members of Wellcome Sanger Institute Scientific Operations: DNA Pipelines collective are listed here: https://doi.org/10.5281/zenodo.5746904. Members of the Tree of Life Core Informatics collective are listed here: https://doi.org/10.5281/zenodo.6125046. Members of the Darwin Tree of Life Consortium are listed here: https://doi.org/10.5281/zenodo.6418363.
