# Supplementary material for: The genome sequence of the garden grass-veneer, Chrysoteuchia culmella (Linnaeus, 1758)
Source: Wellcome Open Res. 2022 Oct 5;7:248. [Version 1] doi: 10.12688/wellcomeopenres.18107.1 (PMC10011750; doi:10.12688/wellcomeopenres.18107.1)
Supplement: Peer review history

# Peer review: Reviewer response for version 1

**DOI:** 10.21956/wellcomeopenres.20078.r54963

**Published:** 2023-03-13

**Authors:** Shuai Zhan

**Affiliations:** 1 University of the Chinese Academy of Sciences,, Beijing, China

In this manuscript, the authors report a genoe reference for the garden grass-veneer moth, *Chrysoteuchia culmella.* The genome reference was assembled in a high level of contiguity and fully anchored to 31 chromosomes. The mitochondrial genome was also assmbled in a reasonable length. Overall, the manuscript was clearly written. I have only concern in the method, in which the authors did not provide detailed information and some of them were simply given a previous reference. No other audience could repeat the assembly with currently provided methods. Also, it's unclear to my how chromosome Z was assigned.

Are sufficient details of methods and materials provided to allow replication by others?

No

Is the rationale for creating the dataset(s) clearly described?

Yes

Are the datasets clearly presented in a useable and accessible format?

Yes

Are the protocols appropriate and is the work technically sound?

Yes

Reviewer Expertise:

Genomics; Evolution.

I confirm that I have read this submission and believe that I have an appropriate level of expertise to confirm that it is of an acceptable scientific standard, however I have significant reservations, as outlined above.

# Peer review: Reviewer response for version 1

**DOI:** 10.21956/wellcomeopenres.20078.r54975

**Published:** 2023-03-13

**Authors:** Ma Cristina Del Rincón-Castro

**Affiliations:** 1 Universidad de Guanajuato,, Campus Irapuato-Salamanca, Mexico

The article deals with genome assembly from a male Chrysoteuchia culmella (the garden grass-veneer; Arthropoda; Insecta; Lepidoptera; Crambidae). The authors report that the genome sequence is 645 megabases in span. The majority of the assembly (99.81%) is scaffolded into 31 chromosomal pseudomolecules with the Z sex chromosome assembled. The article presents the complete information for a database of genomic data on this insect, its approval is recommended.

Are sufficient details of methods and materials provided to allow replication by others?

Yes

Is the rationale for creating the dataset(s) clearly described?

Yes

Are the datasets clearly presented in a useable and accessible format?

Yes

Are the protocols appropriate and is the work technically sound?

Yes

Reviewer Expertise:

Molecular biology of baculoviruses as biological control agents

I confirm that I have read this submission and believe that I have an appropriate level of expertise to confirm that it is of an acceptable scientific standard.

# Peer review: Reviewer response for version 1

**DOI:** 10.21956/wellcomeopenres.20078.r52686

**Published:** 2022-11-17

**Authors:** Théo Léger, Josh Penalba

**Affiliations:** 1 Museum für Naturkunde Berlin, Leibniz-Institut für Evolutions- und Biodiversitaetsforschung Invalidenstr, Berlin, Germany; 2 Museum für Naturkunde Berlin, Leibniz-Institut für Evolutions- und Biodiversitaetsforschung Invalidenstr, Berlin, Germany

This short and concise data-release article provides a complete genome of the garden grass-veneer. The molecular and data analysis methods look sound to me (but as I am no expert in genomics I leave this part to my colleague). I would only have two comments here:

1. Identification of the specimens should be confirmed through blasting the COI DNA barcode against BOLD or GenBank, as worn specimens could be misidentified (similar to Thysanotia, Chrysocrambus or some Catoptria). It looks to me as C. culmella indeed but a double-check is worth it.
2. The "genome annotation" part is a bit short, and it is not stated whether all genes the Ensembl gene set were found in the present genome. This part could be completed for the reader.

Here are the comments of the co-reviewer:

1. Page 4. Col. 1. “While not fully phased, the assembly deposited is of one haplotype” – This isn’t entirely clear. Does this mean for SNPs which were not phased a random allele was chosen for the haplotype?
2. Page 5. Col. 1. “Fragment size analysis of 0.01-0.5ng of DNA…” The placement of this statement is not clear because it precedes the DNA extraction. Is there another source of DNA this is referring to or should this be later?
3. Page 8. Col1. Genome assembly. “Assembly was carried out with Hifiasm” Specify that the only data that was used here was the PacBio HiFi and the 10X Genomics was only used for the polishing.

We both agree that the manuscript can be considered for indexing once these points are addressed.

Are sufficient details of methods and materials provided to allow replication by others?

Yes

Is the rationale for creating the dataset(s) clearly described?

Yes

Are the datasets clearly presented in a useable and accessible format?

Yes

Are the protocols appropriate and is the work technically sound?

Yes

Reviewer Expertise:

I work on the systematics and phylogeny of pyraloid moths

We confirm that we have read this submission and believe that we have an appropriate level of expertise to confirm that it is of an acceptable scientific standard.
